# 5-hydroxymethylcytosine but not MTAP methylation status can stratify malignant pleural mesothelioma based on the lineage of origin

**DOI:** 10.1186/s40248-018-0137-4

**Published:** 2018-08-02

**Authors:** Matteo Bosio, Elena Salvaterra, Francesca Datturi, Patrizia Morbini, Michele Zorzetto, Simona Inghilleri, Stefano Tomaselli, Patrizia Mangiarotti, Federica Meloni, Isa Cerveri, Giulia Maria Stella

**Affiliations:** 1 IRCCS Fondazione Policlinico San Matteo- Unit of Respiratory System Diseases, University of Pavia Medical School, Piazzale Golgi 19, 27100 Pavia, Italy; 2 IRCCS Fondazione Policlinico San Matteo- Pathology Unit, University of Pavia Medical School, Pavia, Italy

**Keywords:** Malignant pleural mesothelioma, Cancer, Epigenetics, Methylation, Lineage of origin

## Abstract

**Background:**

Malignant pleural mesothelioma (MPM) is an aggressive tumor with poor prognosis, mainly associated with work or environmental exposure to asbestos. MPM’s molecular profile is largerly unexplored and effective therapies are still lacking. MPM rarely harbours those somatic genetic lesions that usually characterize solid epithelial-derived tumors. On this basis, our study aims at investigating MPM epigenetic profile.

**Methods:**

We here assessed through immunohistochemistry, FISH and methylation specific PCR, the expression of 5-hydroxymethylcytosine (5- hmC) - an epigenetic marker and an important regulator of embryonic development and carcinogenesis - and the methylation status of the promoter of the MTAP gene - encoding for an enzyme involved in the rescue process of methionine and adenine - in two relevant series of FF-PE MPM samples derived from MPM thoracoscopic biopsies. Tissue sampling was performed at diagnosis.

**Results:**

Within the limitations of the study cohort, the 5-hmC immunophenotype was different among the histological MPM types analysed. In fact, 18% of the epithelial MPMs were negative, 47% weakly positive, and 35% of the cases showed an intense expression of 5-hmC. Sarcomatoid and biphasic MPMs showed intense 5-hmC expression pattern (positive and weakly positive in more than 80% of cases). Among MPM featuring epithelial lineage, none showed methylation of MTAP promoter.

**Conclusions:**

Mesothelial sarcomatoid tumors featured a methylation profile characterized by a permanent gene silencing. Epithelial MPM methylation profile was in-between that of sarcomatoid MPM and the one of epithelial-derived tumors. MTAP promoter methylation level cannot be considered a suitable biomarker of epithelial MPM arousal.

## Background

Malignant pleural mesothelioma (MPM) is an aggressive tumor which arises from pleural layer that is characterized by resistance to conventional treatment modalities and poor prognosis [1]. In the majority of cases, MPM is associated with work or environmental exposure to asbestos fibers [1, 2]. Importantly, it can occur after a long latency [3]. The incidence of MPM is increasing and is expected to reach its peak by 2020 [4]. MPM’s molecular profile is almost unknown so that the disease is still lacking effective therapeutic prospects. Recently, it has been shown that germline *BAP1* mutations are rare events that might predispose to MPM. Furthermore, somatic *BAP1* changes are frequently reported [5], followed by mutations in *NF2* (encoding for merlin) and *CDKN2A* (encoding for p16^INK4A^ and p14^ARF^). Comprehensive genomic analysis allowed the identification of recurrent gene fusions and splice alteration as frequent mechanisms of inactivation of *NF2*, *BAP1* in MPM and reported alterations in Hippo, mTOR, histone methylation RNA helicase and p53 signaling pathways [6]. Transcriptomic analysis demonstrated that poorest prognosis is associated to the activation of the epithelial-to-mesenchymal transition program which mainly affects sarcomatoid subtypes [7]. Recent insight in regarding epigenetic alterations in MPM showed that they are common events during disease onset and progression [8]. A better understanding of epigenetic mechanisms affecting MPM is, thus, mandatory to provide novel therapeutic opportunities against MPM.

On this basis, our study aimed at investigating in two relevant cohorts of MPM the expression of 5-hydroxymethylcytosine (5- hmC), an epigenetic marker and an important regulator of embryonic development and carcinogenesis [9]. Moreover, we investigated the methylation status of the promoter of the methylthioadenosine phosphorylase (*MTAP*) gene, encoding for an enzyme involved in the rescue process of methionine and adenine. Inactivation of this gene – which is known to be involved in oncogenesis of different malignancies – may occur through two different mechanisms: i) genetic deletion; ii) hypermethylation of the promoter. Many solid tumours and hematologic malignancies lack expression of the MTAP enzyme, due to either deletion of the *MTAP* gene or methylation of the *MTAP* promoter. Solid tumors frequently lacking MTAP include MPM, non-small cell lung cancer (NSCLC), gliomas and pancreatic cancer. The hypermethylation of *MTAP* promoter is also involved in hepatocellular carcinoma as well as gastric adenocarcinoma onset [10, 11]. *MTAP* is located at the *INK4* locus near the tumour suppressor gene p16^INK4A^. Homozygous deletion of *CDKN2A* (p16) is one of the most common genetic alterations in pleural mesotheliomas, occurring in up to 74% of cases [12, 13]. *MTAP* resides in the same gene cluster of the 9p21 region and is co-deleted in the majority of *CDKN2A* deleted cases (90%) [11, 12, 14]. Within regard to MPM, it has been recently reported that *MTAP* is frequently deleted. The combination of MTAP and BPA1 expression levels, detected by immunohistochemistry, appears to be a reliable and useful method for differentiating MPM cell from reactive mesothelial cells [15] with a good sensitivity and 100% specificity in detecting MPM [16].

## Methods

### Cases identification and selection

A total of forty formalin-fixed paraffin-embedded (FFPE) samples derived from thoracoscopic biopsies of MPM patients was consecutively retrieved from the archives of the Pathology Division of the IRCCS Fondazione Policlinico San Matteo Hospital. For each case, exhaustive clinical data were also available. The study received ethical approval from local institutional review boards. Out of the 40 cases, 10 were female (25%) and 30 (75%) were male; the mean age at diagnosis was 67,57 ± 9,03 years. Out of them - accordingly to pathologic diagnosis - 15 cases were sarcomatoid MPM, 7 biphasic types and the remaining 18 cases were epitheliod tumors. Six out of the 40 analyzed patients reported a proved work exposure to asbestos fibers; overall the vast majority of cases (95%-38 patients) referred environmental exposure. All patients featured advanced disease and underwent conventional chemotherapy (platinum/pemetrexed) as first line approach. The overall survival of the analysed cohort was 14.87 months (st: ± 9.40). Clinical data are listed in detail in Table 1.

**Table 1:** Clinical and demographic data of the analysed cohort

	Patient ID	Gender	Age at diagnosis	Histology	Exposure to asbestos	TNM stage	Therapy	OS (months)
1	M01	M	47	E	Work	IV	S + C	7
2	M02	M	77	S		IV	S + C	13
3	M03	M	70	E		IV	C	43
4	M04	M	57	S	Work	IV	C	6
5	M05	M	70	E	Work	IV	S + C	11
6	M06	F	70	B		IV	C	8
7	M07	M	72	S		IV	C	10
8	M08	M	64	E		IV	S + C	5
9	M09	F	66	E		IV	C	18
10	M10	F	71	E		IV	C	4
11	M11	M	78	S		IV	C + R	13
12	M12	M	76	E		IV	C	5
13	M13	F	70	E	Work	IV	C	7
14	M14	M	72	S		IV	C + R	15
15	M15	F	77	S		IIIB	S + C	21
16	M16	M	75	E		IV	C	9
17	M17	M	70	S		IV	C	15
18	M18	F	76	E		IIIB	S + C	24
19	M19	M	72	B		IV	S + C	7
20	M20	M	80	E		IV	S + C	10
21	M21	M	74	S		IV	S + C	18
22	M22	M	60	B		IV	S + C	26
23	M26	M	57	S		IV	S + C	19
24	M29	M	64	S		IV	C	19
25	M32	M	74	E		IV	C + R	26
26	M33	M	71	B		IV	S + C	6
27	M34	M	75	E		IV	S + C	26
28	M35	M	60	S		IV	S + C	47
29	M37	M	57	E		IV	S + C	16
30	M41	M	44	B		IV	S + C	7
31	M42	M	60	S		IV	S + C	13
32	M45	M	73	E		IV	S + C	12
33	M47	M	68	S		IV	S + C	15
34	M48	F	58	E		IIIB	S + C	7
35	M77	M	56	B		IV	C	6
36	M78	F	80	S	Work	IV	S + C	15
37	M79	F	72	E		IV	S + C	19
38	M80	M	70	B		IV	S + C	20
39	M81	M	48	S		IV	S + C	16
40	M84	F	63	E	Work	IV	S + C	11

E stands for epitheliod MPM, S: sarcomatoid, B: biphasic types; S stands for surgery, C for chemotherapy, R for radiotherapy

A second cohort, represented by a series of ninety FFPE blocks from surgical biopsies epithelial MPM, was available as well.

In all cases, tissue sampling was performed at diagnosis (before the beginning of chemo- and radiotherapy).

### Cytogenetic analysis

#### Immunohistochemistry (IHC)

Immunohistochemical analysis as well as fluorescence in situ hybridization study have been performed in order to correlate protein expression and homozygous deletion in mesothelioma tissues. The immunohistochemical protocol for 5-mhC staining used in this report was performed according to the previously optimized and validated method by Haffner et al. [17]. The 5-hmC staining intensity was scored as none (0), weak (1), moderate (2) or marked (3), according to Lian et al. [18]. In detail 0 = no immunolabeling; + = less intense than immunolabeling of in adjacent benign cells; ++ = comparable with normal nuclei; and +++ = more intense than normal nuclei.

Immunostaining for MTAP was performed according to Kinoshita et al. [14] and scored as follows: very strong expression (+++), strong expression (++) low level of expression (+). As a control, we checked the expression of 5-hmC and MTAP in a series of biopsies from reactive mesothelial hyperplasia (RMH) samples as well as from different proliferative lung pathologies: inflammatory conditions (idiopathic pulmonary fibrosis (IPF / UIP) and cryptogenic organizing pneumonia (COP), and cancer (adenocarcinoma (ADC), squamous-cell carcinoma (SCC)) and healthy lung tissue samples obtained from lobectomies performed to resect tumor masses.

#### Methylation specific PCR and FISH analysis

The method used to quantify promoter MTAP hyper methylation was sensitive melting analysis after real-time methylation specific-PCR (SMART-MSP), a diagnostic tool that permits to quantify the methylation levels of genes considered promising DNA methylation biomarkers for early cancer diagnostics. The methylation specific PCR has been conducted as already published [19]. The FISH was performed on the entire INK4 locus was performed on 4-μm-thick tissue cell block sections as previously described [14].

Details for both methods are described in the Supplementary Material Section.

### Statistical analysis

Five-hmC scores were analyzed as interval data sets using two-sided Student’s t-test. A *p* ≤0.05 was considered statistically significant. Kappa statistics were used to assess the correlation between IHC expression of MTAP and homozygous deletion status of 9p21 FISH in cell blocks. All statistical analyses were performed using R statistical software (version 3.2.2; R Foundation for Statistical Computing, Vienna, Austria).

## Results

### 5-hmC expression

Out of the 18 biopsies related to epithelioid MPM, 13 featured negative expression and in 5 cases an intense expression was revealed. The sarcomatoid histotypes and biphasic ones showed a more intense pattern of expression, higher than 50% of the cases of each group, compared to those with an epithelioid histotype (Table 2). Obviously, the small number of samples carrying sarcomatoid and biphasic histotype does not allow a more extensive conclusion; however, a clear prevalence of a more intense expression might be featured by tumors with mesenchymal lineage of origin.

**Table 2:** Results of 5-hmC immunohistochemistry analysis

5-hmC IHC		Positive	Negative
MPM	E	5	13
	S	9	6
	B	5	2
RHM		6	19
COP		5	15
NSCLC	ADC	0	25
	SCC	0	25
IPF		0	15
Healthy lung		0	25
Sensitivity MPM/RMH = 47%			
Specificity MPM/RMH = 76%			
Sensitivity E/B + S = 63%			
Specificity E/B + S = 72%			

E stands for epitheliod MPM, S: sarcomatoid, B: biphasic types

As control, we considered the expression of 5-hmC in a series of RMH: none of them harbored positive staining. Moreover, we selected pulmonary biopsies of patients with idiopathic pulmonary fibrosis (IPF/UIP), cryptogenic pneumonia in organization (COP), adenocarcinoma (ADC), squamous cell carcinoma (SCC) and in healthy lung tissue samples. A general positivity at 5-hmC was reported in samples of healthy lungs and COP. We documented both in the ADCs and in the SCCs observed a negative/weak marker expression. The 25% of the IPF/UIP samples showed a pattern like that of healthy controls and COP, characterized by a strong expression in the bronchial and alveolar epithelial cells. The remaining 75% of the fibrotic samples showed a moderate/negative expression in the bronchiolar cells, in the cells of the terminal bronchioles and in the pneumocytes of type II activated (data not shown). Overall, the analysis documented a low sensitivity of the analysis (47%) in distinguishing MPM and RMH whit an acceptable specificity (76%). Within respect to MPM the test displayed a good sensitivity (63%) and specificity (72%) in stratifying cases based on the lineage of origin (Table 2).

### MTAP expression

**Fig. 1:**
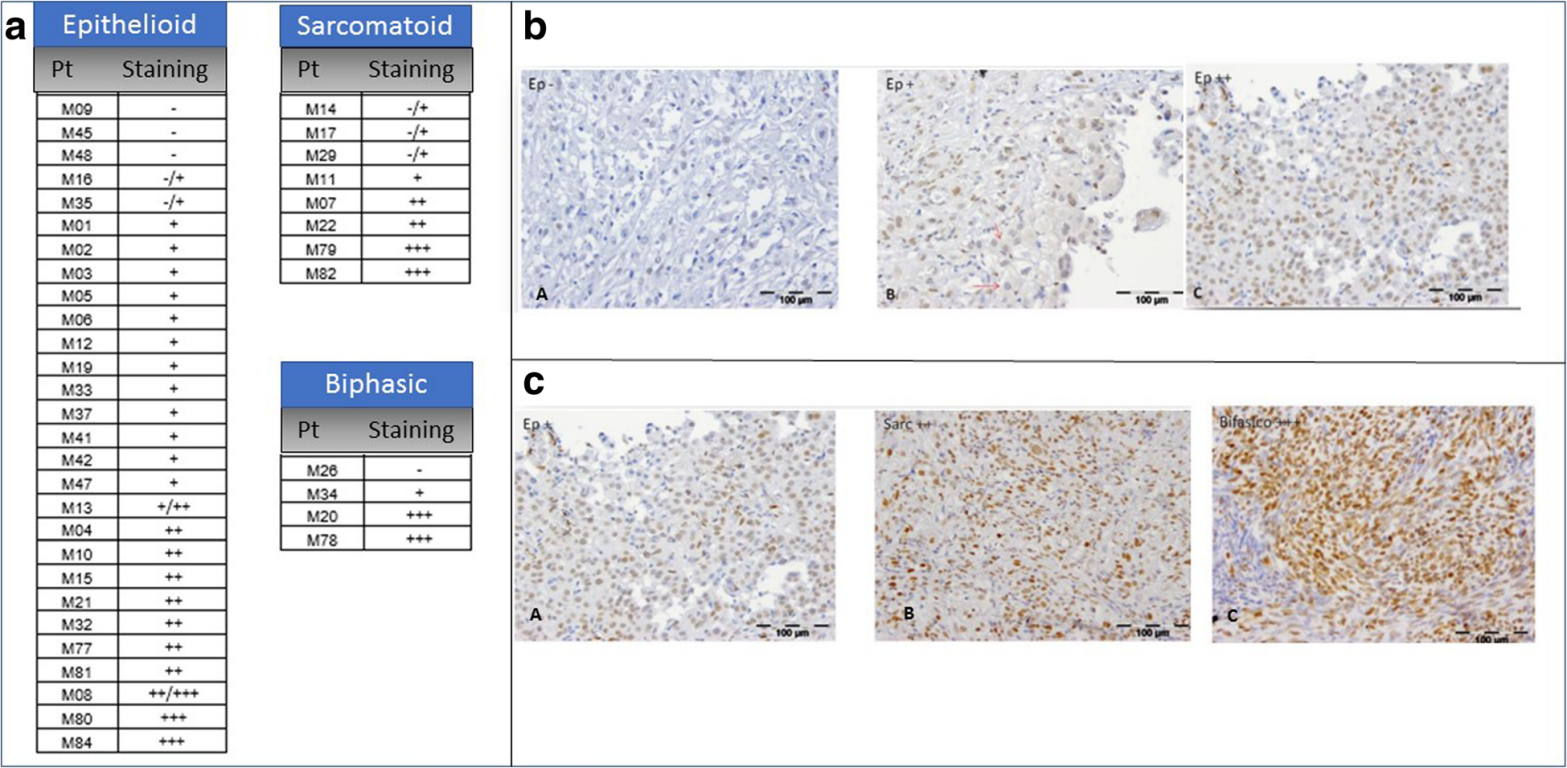
Panel **A**. Immunohistochemistry staining for 5-mhC: semiquantitative score for each case and subtype. Panel **B.** Different levels of 5-mhC expression by IHC among epitheliod mesothelioma samples. A: negative; B weakly positive; C: highly positive. Panel **C.** Different levels of 5-mhC expression by IHC among mesothelioma subtypes. A: weakly positive epitheliod MPM, B: intensely positive sarcomatoid MPM; C: strong intensely positive biphasic MPM type, featuring prevalence of sarcomatoid cells

**Fig. 2:**
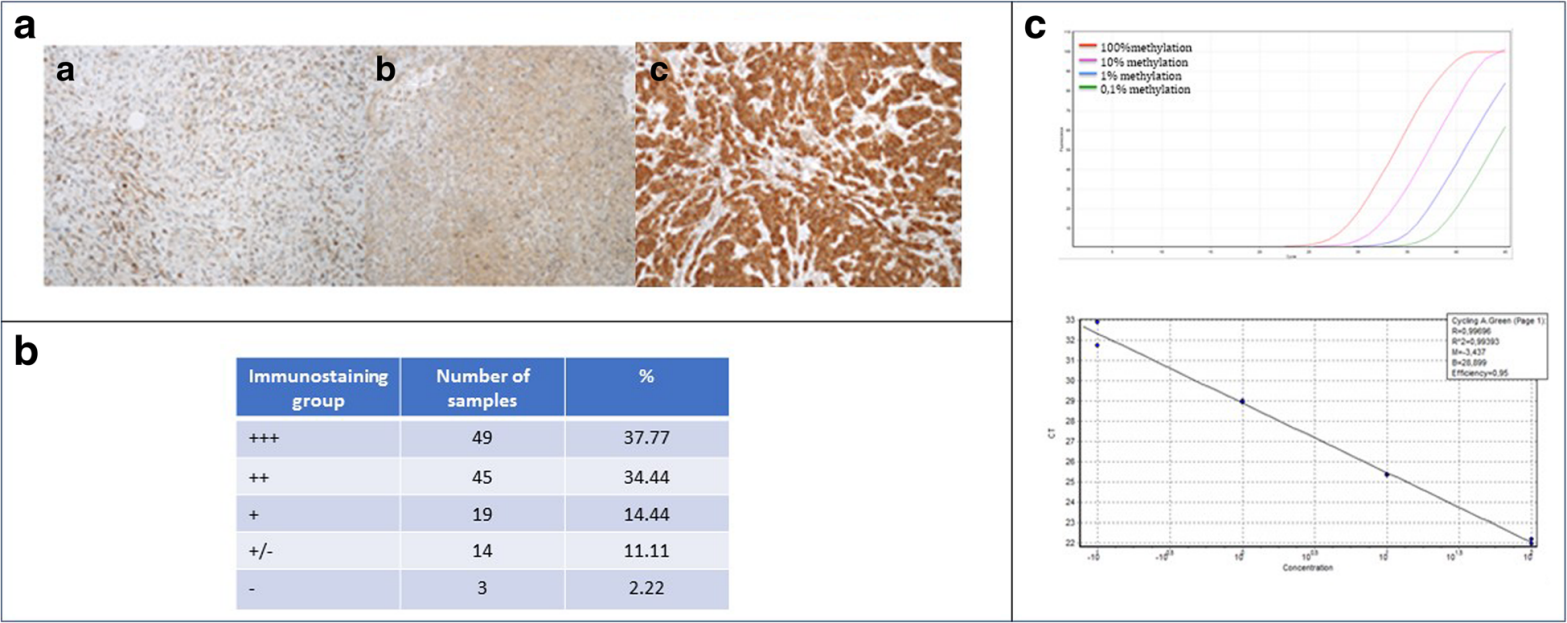
Panel **A**. Immunohistochemistry staining for INK4 locus. A. INK4 locus immunohistochemistry staining of sample 4, group +/−. B. INK4 locus immunohistochemistry staining of sample 3, group ++. C. INK4 locus immunohistochemistry staining of sample 47, group +++. Panel **B**. Immuno-staining groups and relative percentages (40 + 90 cases analyzed). Panel **C**. Sensitivity and quantitative accuracy of MTAP SMART-MSP assay (90 cases analyzed). Top: The assay was sensitive to 0,1% methylation in a background of WGA. Bottom: The assay was quantitative accurate in the range 100% methylation to 0,1% methylation (R2 = 0,99,393). The PCR efficiency was 0,95

One hundred and thirty (130) samples were checked for MTAP expression by immunohistochemistry staining. The cohort derived from the previously described one (40 cases) and a series of ninety epithelioid MPM. Out of them, only two samples (2,22%) were negative for MTAP expression. Some tumours contain more intensities as +/− indicating that samples showed different degrees of MTAP expression even though in some cases the staining was weak. Four main groups were defined. The first group consisted of samples that expressed MTAP protein very strongly (+++), the second contained samples that expressed MTAP strongly (++) and the third comprised samples that expressed MTAP at low level (Fig. 1.). Finally, samples showing a mix between cells expressing MTAP and cells lacking it, belonged to the fourth group (+/−). To be assessed as MPM, the cells had to be positive for the two MPM markers: calretinin and KL1 [20]. We had available 89 fluorescence in situ hybridization results from MPM samples even if 6 out of them showed no data. Heterozygous deletion of the locus INK4 was not observed in any MTAP positive tumours. Sixty-four (64) samples showed no deletion confirming the IHC results, while 19 of them presented homozygous deletion. The results of the SMART-MSP assay revealed that no one of the samples analysed was methylated (Fig. 2).

What was difficult to explain is why there were 16 FISH results (19,5%) that showed homozygous deletion of the locus *INK4* even if the MTAP protein was expressed. One of the reasons could be due to experimental issues. The FISH probe, in fact, was made of small complementary DNA fragments and it was not a large fragment that span the entire locus INK4. This led to the fact that, even the MTAP was not deleted but p16 was, the signal was too weak to be detected by the fluorescence microscopy. From the Real-time PCR we knew that there was a template because the results showed that there was amplification of MTAP. Another possible explanation could be hemyzygous deletion not detected by the FISH analysis. A previous IHC study reported a complete loss of immunoreactivity in only 19% of the tumours, whereas 45% had some degree of retained protein expression in a cohort of 99 MPMs [21]. They hypothesized that some tumours may only harbour a hemizygous deletion rather than the homozygous deletion leading to complete protein loss. From these evidences we can assess that in the sequence in between the two genes *MTAP* and *p16* there is a breakpoint region more prone to be deleted. Investigating the database Gene (NCBI) we knew that this region could be *LOC100533725 HERV-FRD* provirus ancestral Env polyprotein pseudogene. Moreover, the use of dual-colour probes during the FISH analysis could give false positive results for deletions. We could not confirm the presence of false positive results for deletion in the FISH analysis, neither the existence of a breakpoint region between *MTAP* and *p16.* Our results indicated that *MTAP* promoter hypermethylation seemed not to be involved in MPM carcinogenesis and that the *MTAP* and *p16* co-deletion was present in none of the analysed 90 MPM samples. Overall, we could conclude that MTAP hypermethylation might not behave as a good biomarker for malignant mesothelioma.

## Discussion

Although genomic alterations play a driving role, more recent evidence shows that changes that are not directly implicated in the DNA sequence also play an important role in cancer development. These epigenetic modifications affect temporal and spatial control of gene activity required for homeostasis of complex organisms. The global epigenetic profile determined by high-throughput methylation analysis differs between MPM and normal pleura, indicating that MPM, like other cancers, has aberrant CpG island methylation [7]. Globally these data suggested that the expression level of 5-hmC is significantly reduced in human tumors and this is consistent with the complexity of the epigenomic alterations that characterize malignant proliferation. Furthermore, the depletion of 5-hmC - detected by immunohistochemical analysis - can constitute a biomarker usable in the diagnosis of cancer. It is well documented that in MPM – differently from solid tumors of epithelial origin – known somatic alterations activating oncogenes or inactivating tumor suppressors are rarely found [5, 22]. It has been thus further investigated the epigenetic profile characterizing MPM. Even with the limits of the study cohort, immunohistochemical analyses aimed at documenting the level of 5-hmC expression showed conflicting results in different MPM subtypes with globally low expression in epithelioid forms versus higher levels in the sarcomatoid ones. Notably, results are not affected by MPM exposure to chemo agents and/or ionizing radiation. Therefore, it seems probable to conclude that the different lineage of origin can play a role in the methylation status of the different tumor subtypes. Thus, those tumors that originate from mesodermal derived cells (mesothelioma, mainly the sarcomatoid histotype) have an ‘atypical’ methylation profile characterized by elevated levels of 5- hmC. To deeper investigate these data, the MTAP expression was analyzed in a parallel cohort of 90 epithelioid MPM samples. The methylation status of the *MTAP* promoter was studied by designing a specific primer for the SMART-MSP assay, in order to quantify the methylation level of biopsy samples. Unexpectedly, none of the 90 samples of MPM analyzed shows methylation of the MTAP promoter. These results, although preliminary, suggested that hypermethylation of the *MTAP* promoter seems not to be involved in the onset of MPM featuring epithelial lineage. It can therefore be concluded that the latter cannot be considered a suitable biomarker to determine epithelial MPM onset and progression. An open problem remains linked to the need for defining the exact starting sequence of the deletion between *MTAP* and *p16*. Overall, the results of this screening are consistent with the extreme biomolecular heterogeneity that characterizes MPM and explain the complexity of the approaches required for a more in-depth definition of the pathogenetic mechanisms and of targeted therapeutic options.

## Conclusions

Overall, a prevalence of sarcomatoid mesotheliomas showed a methylation profile recalling what has been called *‘epigenetic cancer stem cell signature’*, characterized by a permanent gene silencing, which favors the stay of the cell in one self-renewal state, predisposing it to malignant transformation [23]. In this global framework, the absence of MTAP promoter methylation is likely to be related to the fact that the gene is silenced primarily as a result of deletion, as already reported in the literature. The epitheliod mesotheliomas instead show a profile straddling the previous one and the one typical of epithelial-derived tumors, characterized by 5-hmC depletion. A second key point is linked to the need for a more in-depth characterization of the molecular mechanisms through which asbestos nanofibers might affect gene expression, even including methylation status by their direct interaction with chromatin.

## Abbreviations

5-hmC: 5-hydroxymethylcytosine
ADC: Adenocarcinoma
COP: Cryptogenic organising pneumonia
FFPE: Formalin fixed paraffin embedded
IPF: Idiopathic pulmonary fibrosis
MPM: Malignant pleural mesothelioma
*MTAP*: Methylthioadenosine phosphorylase
SCC: Squamous cell carcinoma
